# Enhanced endothelial motility and multicellular sprouting is mediated by the scaffold protein TKS4

**DOI:** 10.1038/s41598-019-50915-5

**Published:** 2019-10-07

**Authors:** Elod Mehes, Monika Barath, Marton Gulyas, Edina Bugyik, Miklos Geiszt, Arpad Szoor, Arpad Lanyi, Andras Czirok

**Affiliations:** 1 Department of Biological Physics, Eotvos University, Budapest, Hungary; 2 Department of Immunology, Faculty of Medicine, University of Debrecen, Debrecen, Hungary; 3 First Department of Pathology and Experimental Cancer Research, Semmelweis University, Budapest, Hungary; 4 Department of Physiology, Faculty of Medicine, Semmelweis University, Budapest, Hungary; 5 Department of Biophysics and Cell Biology, Faculty of Medicine, University of Debrecen, Debrecen, Hungary; 6 Department of Anatomy & Cell Biology, University of Kansas Medical Center, Kansas City, KS USA

**Keywords:** Cellular motility, Cell migration, Cellular imaging, Image processing, Angiogenesis

## Abstract

Endothelial cell motility has fundamental role in vasculogenesis and angiogenesis during developmental or pathological processes. Tks4 is a scaffold protein known to organize the cytoskeleton of lamellipodia and podosomes, and thus modulating cell motility and invasion. In particular, Tks4 is required for the localization and activity of membrane type 1-matrix metalloproteinase, a key factor for extracellular matrix (ECM) cleavage during cell migration. While its role in transformed cells is well established, little is known about the function of Tks4 under physiological conditions. In this study we examined the impact of Tks4 gene silencing on the functional activity of primary human umbilical vein endothelial cells (HUVEC) and used time-lapse videomicrosopy and quantitative image analysis to characterize cell motility phenotypes in culture. We demonstrate that the absence of Tks4 in endothelial cells leads to impaired ECM cleavage and decreased motility within a 3-dimensional ECM environment. Furthermore, absence of Tks4 also decreases the ability of HUVEC cells to form multicellular sprouts, a key requirement for angiogenesis. To establish the involvement of Tks4 in vascular development *in vivo*, we show that loss of Tks4 leads sparser vasculature in the fetal chorion in the Tks4-deficient ‘nee’ mouse strain.

## Introduction

Cell motility is essential in the morphogenic events of embryonic development and subsequent ontogenesis. Vascular network formation in warm-blooded vertebrates is an emergent process in which migration of vascular precursor cells is of key importance^1–3^. This first phase of vascular network formation, termed primary vasculogenesis, is best studied in birds due to their ease of access for experiments and similarities with the vascularization of murine embryos^4,5^. In avian embryos, endothelial precursors differentiate in a spatially scattered manner in the lateral mesoderm or within aggregates in the extraembryonic mesoderm. Angioblasts then engage in active motion within the extracellular matrix (ECM) environment and self-assemble into a polygonal network before the onset of circulation^1,6–8^. In the subsequent phase of vascular network formation, termed angiogenesis, endothelial cells form tubes carrying blood and the initial vascular network is further reshaped by vessel sprouting (outgrowth of new vessels from existing ones), fusion or withdrawal as instructed by surrounding tissues and haemodynamic forces. Angiogenesis also involves the collective migration of endothelial cells^9,10^, and it occurs throughout life in various physiological and pathological conditions, including the vascularization of tumors^11–13^. Understanding the mechanisms of angiogenesis is also a key factor in vascularization of engineered tissues^14–17^. Studying angiogenic processes in suitably simplified 2D and 3D *in vitro* models, which we have extensively utilized so far, can yield relevant information about their characteristic features and dynamics.

Tks4/SH3PXD2B was discovered as a transcript activated in early adipogenesis^18^, based on its homology to the cytoskeleton organizer protein Tks5^19^ and to the NADPH oxidase/NOX2 organizer p47phox^20^. Subsequent studies identified Tks4 as one of the key organizers of the cytoskeletal structure in podosomes and lamellipodia, and thus essential for appropriate regulation of motility and invasion of transformed cells^19,20^. Tks4 was shown to promote migration of tumor cells in various *in vitro* models and increased the number of metastases in a B16 murine melanoma model^21,22^. Although the molecular scaffold organized by Tks4 has not yet been described in detail, several cytoskeletal proteins that interact with Tks4 have been identified^20,21^. Importantly, both Tks4 and its homologue Tks5 are required^19,22^ for the cell surface expression and hence the activity of the membrane type 1-matrix metalloproteinase (MT1-MMP/MMP14), a master regulator of other MMPs^23^. In the absence of the Tks proteins 3D proliferation of the human melanoma cell line C8161.9 was hampered in 3D collagen I matrix while it was unaffected when seeded on a 2D coating of the same matrix or on plastic surfaces.

Although much has been described about Tks4 as a regulator of cytoskeletal structures in transformed cells, we have relatively little information about its function in healthy cells and tissues. The role of Tks4 in the development of various tissues is well demonstrated by the severe phenotype observed in Tks4-deficient murine models characterized by runted growth, skeletal, eye, and cardiac abnormalities^24,25^. Frank-ter Haar syndrome, the human disease associated with Tks4 mutations is characterized by cranio-facial abnormalities and development of cardiovascular defects resulting in death of the patients at infancy^26^. Another human genetic disease related to mutations in the Tks4 gene is Borrone Dermato-Cardio-Skeletal syndrome, which is also characterized by defective heart development^27^. These defects may develop in part due to impaired cell motility within tissues as a result of defects in cytoskeletal structures.

In this study, as a bottom-up approach, we investigated the motility of two vascular endothelial cell types with silenced Tks4 in different *in vitro* environments and developed a quantitative assay for measuring their vascular sprout growing activity. We show that the lack of functional Tks4 results in defective ECM digestion and decreased cell motility in 3-dimensional ECM, accompanied by diminished vessel sprout growth. As a top-down approach, we studied the morphology of the vasculature in the fetal chorion of Tks4-KO mice and demonstrate that vessel density is below that of wild type at this stage of development. Our results provide an insight to the role of Tks4 in vascular development.

## Results

### TKs4 silencing

Tks4 is expressed by human umbilical vein endothelial cells (HUVEC)^20^ and human cardiac microvascular endothelial cells (HMVEC) used throughout this study in functional assays of cell motility. To directly test the contribution of Tks4 to various endothelial cell functions, we silenced the Tks4 gene in HUVEC and HMVEC cells by transfecting them with Tks4-specific siRNA (termed Tks4-si) while we also used a minimally modified oligonucleotide (termed control-si) as a negative control. Gene silencing resulted in absence of the Tks4 protein in both HUVEC and HMVEC cultures between days 2 and 6 past transfection, as verified by western blot analyses performed on cell lysate samples harvested at various post-transfection time points (Supplementary Figs S1 and S2). Therefore all experiments reported here were performed within this time frame. We also used these lysates to detect Tks5 protein by western blotting. We observed a slight elevation in Tks5 level after silencing the Tks4 gene (Supplementary Fig. S3).

### Gelatin digestion by endothelial cells is decreased by Tks4 silencing

**Figure 1:**
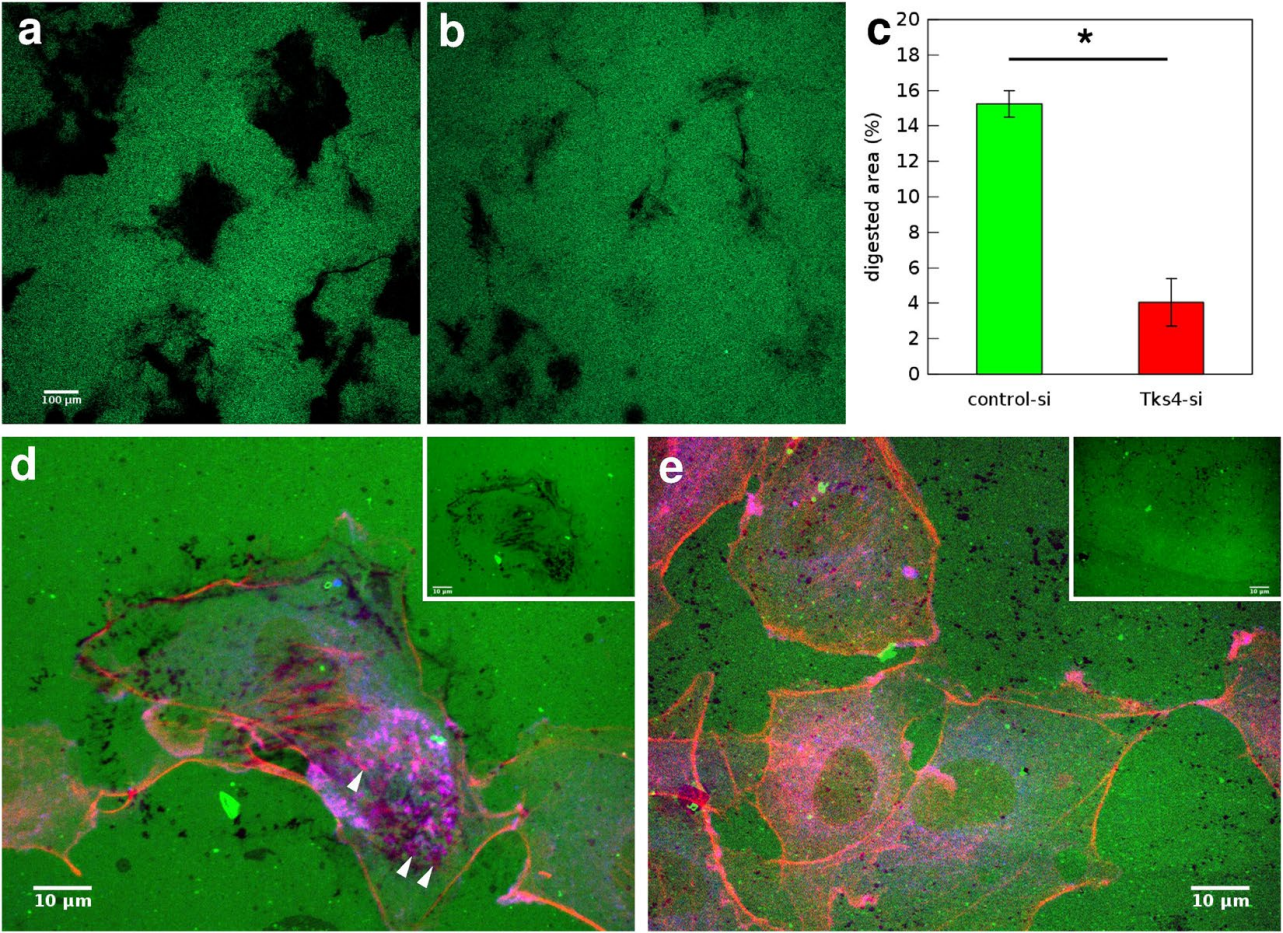
Gelatin degradation assay. (**a**,**b**) Representative fluorescent images of microscopic fields where HUVEC cells transfected with control-siRNA (**a**) or Tks4-siRNA (**b**) and seeded at the same cell density digested fluorescent OregonGreen488-conjugated gelatin substrate for 20 hours. Dark areas correspond to cleaved substrate. Scale bar: 100 *μ*m. (**c**) Quantitative analysis of digested areas from n = 3 independent sets of experiments. Error bars are SEM, asterisk (*) indicates statistically significant difference with Student’s t-test, $p < 0.05$. (**d**,**e**) HUVEC cells transfected with control-siRNA (**d**) or Tks4-siRNA (**e**) and seeded on fluorescent OregonGreen488-conjugated gelatin substrate (green) for 5 hours were immunolabeled with anti-cortactin antibody (blue) and stained with phalloidin-TRITC to visualize F-actin (red). Arrowheads in d point to cortactin positive podosome structures. For better identification of areas with increased proteolytic activity, insets in d and e show the corresponding green fluorescent channel only. Scale bars: 10 *μ*m.

When migrating in the tissue environment cells use matrix metalloproteinases to cleave and remodel the extracellular matrix. Lack of Tks4 in src-transformed fibroblast and melanoma cells was reported earlier to interfere with their ability to secrete and adequately localize matrix metalloproteinases and digest ECM proteins^19,22^. To analyze the effect of Tks4 on ECM degradation by endothelial cells we performed a gelatin degradation assay in our model system of HUVEC cells. Gelatin, a hydrolyzed form of collagen, is cleaved by matrix metalloproteinases among which MT1-MMP takes a leading role^28^. We transfected HUVEC cultures with either control siRNA or Tks4-specific siRNA and seeded them in equal cell density on Oregon Green 488-conjugated gelatin substrate. Cultures were fixed 20 hours after seeding and proteolytic cleavage was detected as a localized loss of fluorescence. Tks4-silenced cells had significantly lower digestion activity as demonstrated by a marked decrease in the size of non-fluorescent areas (Fig. 1).

**Figure 2:**
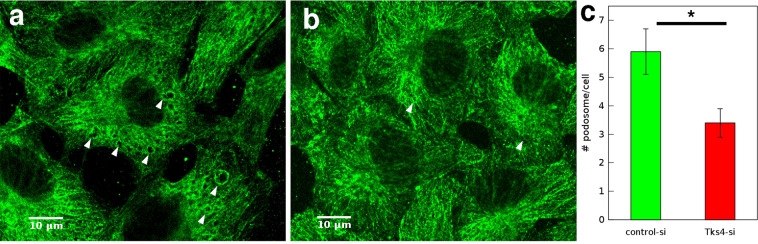
Tks4 increases podosome formation in endothelial cells. (**a**,**b**) Representative MT1-MMP immunofluorescence of HUVEC cells transfected with control-siRNA (**a**) or Tks4-siRNA (**b**) after treatment with 80 nM PMA for 1 h. Arrows point to podosome rosettes. Scale bar: 10 *μ*m. (**c**) Quantitative analysis of podosome rosettes identified in MT1-MMP immunolabeled cultures. Podosomes were counted in n = 91 cells from each group. Error bars indicate SEM, asterisk (*) indicates statistically significant difference with Student’s t-test, $p < 0.05$.

Podosomes are cell membrane protrusion sites with localized metalloproteinase activity required for ECM degradation. Appearance of podosomes and podosome rosette structures can be induced by treatment with phorbol ester (PMA)^29^. To test the impact of Tks4 silencing on podosome formation we immunolabeled MT1-MMP in HUVEC cells. On the basis of MT1-MMP immunolocalization we identified podosome structures and carried out quantitative analysis of podosome density. As expected, Tks4 silencing resulted in considerable reduction in the number of podosomes (Fig. 2). We also seeded these HUVEC cells on Oregon Green 488-conjugated gelatin substrate and induced podosome formation by treatment with 80 nM PMA for 1 hour. Subsequent visualization of cortactin and F-actin, markers of podosome structures^19^ revealed co-localization of cortactin positive podosome structures and sites of matrix degradation (Fig. 1d,e).

### Inactivation of Tks4 does not alter 2D motility of endothelial cells

**Figure 3:**
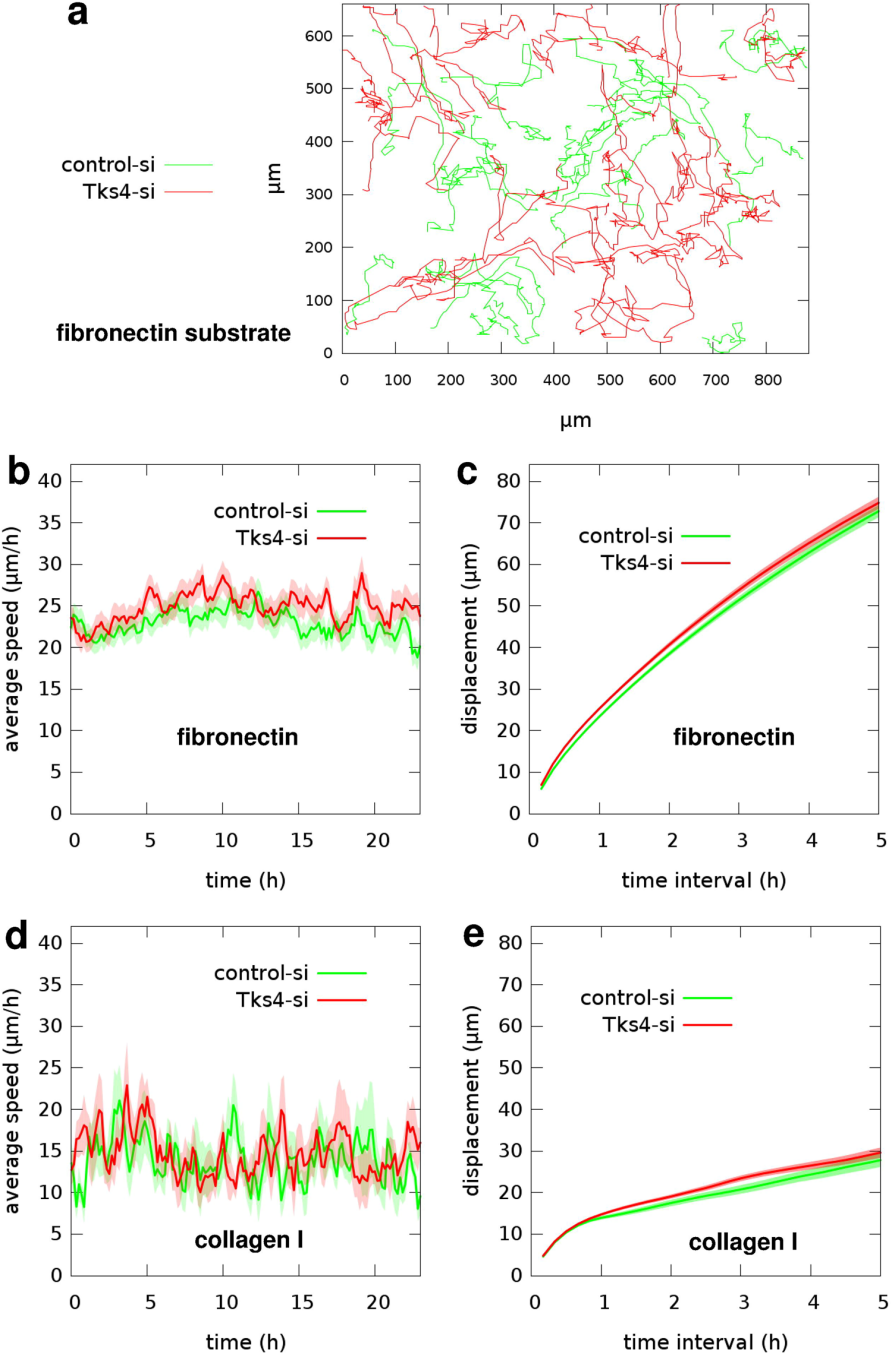
Random motility of endothelial cells on 2D substrates. (**a**) Representative cell trajectories observed in two parallel HUVEC cultures – transfected with control-siRNA (green) or Tks4-siRNA (red) – on fibronectin substrate are superimposed for comparison. Trajectories were extracted from 24 hours long time-lapse recordings, also see Supplementary Videos S1 and S2. (**b**) Time-dependent cell speeds on fibronectin, averaged over the tracked cells for each time point. (**c**) Average cell displacements on fibronectin during various time intervals. Data in (**b** and **c**) were collected from n = 145 control-si cells and n = 151 Tks4-si cells on fibronectin in two independent sets of experiments. (**d**) Time-dependent cell speeds on collagen I. (**e**) Average cell displacements on collagen I. Data in (**d** and **e**) were collected from n = 20 control-si cells and n = 20 Tks4-si cells on collagen I. Error stripes in graphs correspond to SEM.

While random cell motility on a 2D surface requires a sustained cytoskeletal remodeling, it does not involve ECM cleavage. To test the involvement of Tks4 in 2D random motility we seeded Tks4-specific or control siRNA-transfected HUVEC cells on tissue culture plastic surfaces coated with either fibronectin or collagen I. The parallel cultures were recorded by time-lapse videomicroscopy for 24 hours. Subsequently, we tracked cells and analyzed their movement. For the two sets of trajectories we calculated average cell speeds as a function of culture age and average cell displacements for time intervals ranging between 10 minutes and 5 hours. Silencing of Tks4 in endothelial cells did not yield considerable change in either measure of cell motility on fibronectin-coated substrate: The average speed of control siRNA-transfected cells was 23.4 +/− 0.3 *μ*m/h, compared to 25.3 +/− 0.3 *μ*m/h obtained for Tks4-silenced cells. Similarly, we could measure no considerable difference in the motility of parallel HUVEC cultures on collagen I-coated substrate: average speed was 13.9 +/− 0.4 *μ*m/h for control-si, compared to 14.7 +/− 0.3 *μ*m/h for Tks4-si cells (Fig. 3, Supplementary Videos S1 and S2). For a comparison, HMVEC cells transfected with the same siRNAs were tested in a 2D motility assay on fibronectin substrate. Similarly to HUVECs, HMVECs showed no difference in 2D motility regardless of absence of Tks4 (Supplementary Fig. S4, Supplementary Video S3). Additionally, we analyzed the morphology of control-si and Tks4-si HUVEC cells on fibronectin or collagen I substrate. We measured the areas of individual cells using ImageJ software. While cells spread differently on these two substrates and cell areas were larger on fibronectin, we could not detect significant differences in average cell areas between control-si and Tks-si HUVECs on any of these substrates (Supplementary Fig. S5).

### 3D motility of endothelial cells is decreased in the absence of Tks4

**Figure 4:**
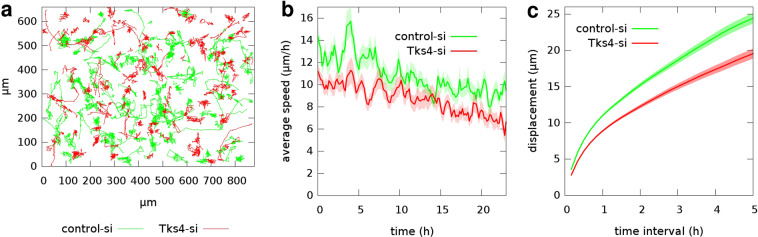
3D random motility of endothelial cells in collagen I gel. (**a**) Representative cell trajectories observed in two parallel HUVEC cultures – transfected with control-siRNA (green) or Tks4-siRNA (red) – are superimposed for comparison. Trajectories were extracted from 24 h long time-lapse recordings, also see Supplementary Video S4. (**b**) Time-dependent cell speeds, averaged over the tracked cells for each time point. (**c**) Average cell displacements during various time intervals. Error stripes correspond to SEM. Data from n = 112 control-siRNA cells and n = 104 Tks4-siRNA cells were collected from two independent sets of experiments.

**Figure 5:**
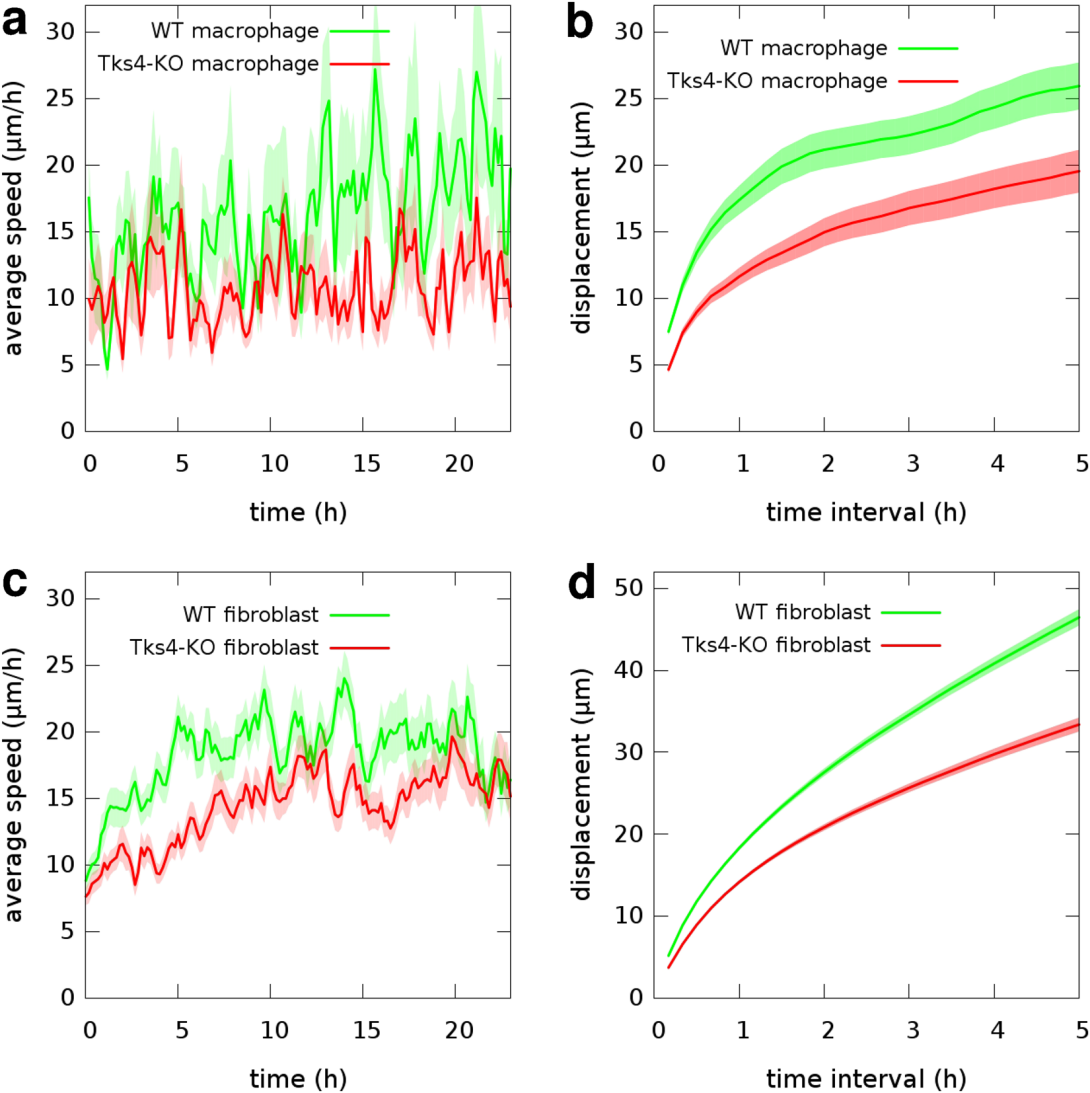
3D motility of macrophages and fibroblasts in collagen I gel. Motility data of macrophages (upper panel) or fibroblast cells (lower panel) isolated from wild type (green) or Tks4-KO (red) mice were collected from 24 hour long time-lapse recordings, also see Supplementary Videos S5 and S6. (**a**,**c**) Time-dependent average cell speeds. (**b**,**d**) Average cell displacements during various time intervals. Error stripes correspond to SEM. Data from n = 41 WT and n = 38 KO macrophages and n = 78 WT and n = 80 KO fibroblast cells were collected from four independent sets of experiments.

Cell migration in 3-dimensional extracellular matrix requires both cleavage of ECM and cytoskeletal remodeling. To study the role of Tks4 in 3D migration we tested the ability of endothelial cells to randomly migrate in collagen I gels after silencing the Tks4 gene. HUVEC cells were seeded within collagen I gels and their motion was recorded for 24 hours by time-lapse videomicroscopy. Cell trajectories extracted from the recorded images were used to calculate time-dependent average cell speeds and average cell displacements for various time intervals (Fig. 4). Tks4-silenced endothelial cells exhibited considerably lower motility: the average speed of Tks4-silenced HUVECs was 8.8 +/− 0.15 *μ*m/h, while control-siRNA-transfected moved with an average speed of 11 +/− 0.16 *μ*m/h. As a comparison, we also tested the 3D motility of primary macrophages and fibroblasts that were isolated from wild type or Tks4 KO (nee) mice and were also seeded within collagen I gels. Similarly to HUVEC, both cell types exhibited lower 3D motility when functional Tks4 was missing (Fig. 5). These results thus suggest a general effect of Tks4 on 3D cell motility, operational in multiple cell types/lineages.

### Multicellular endothelial sprouting is decreased by Tks4 inactivation

**Figure 6:**
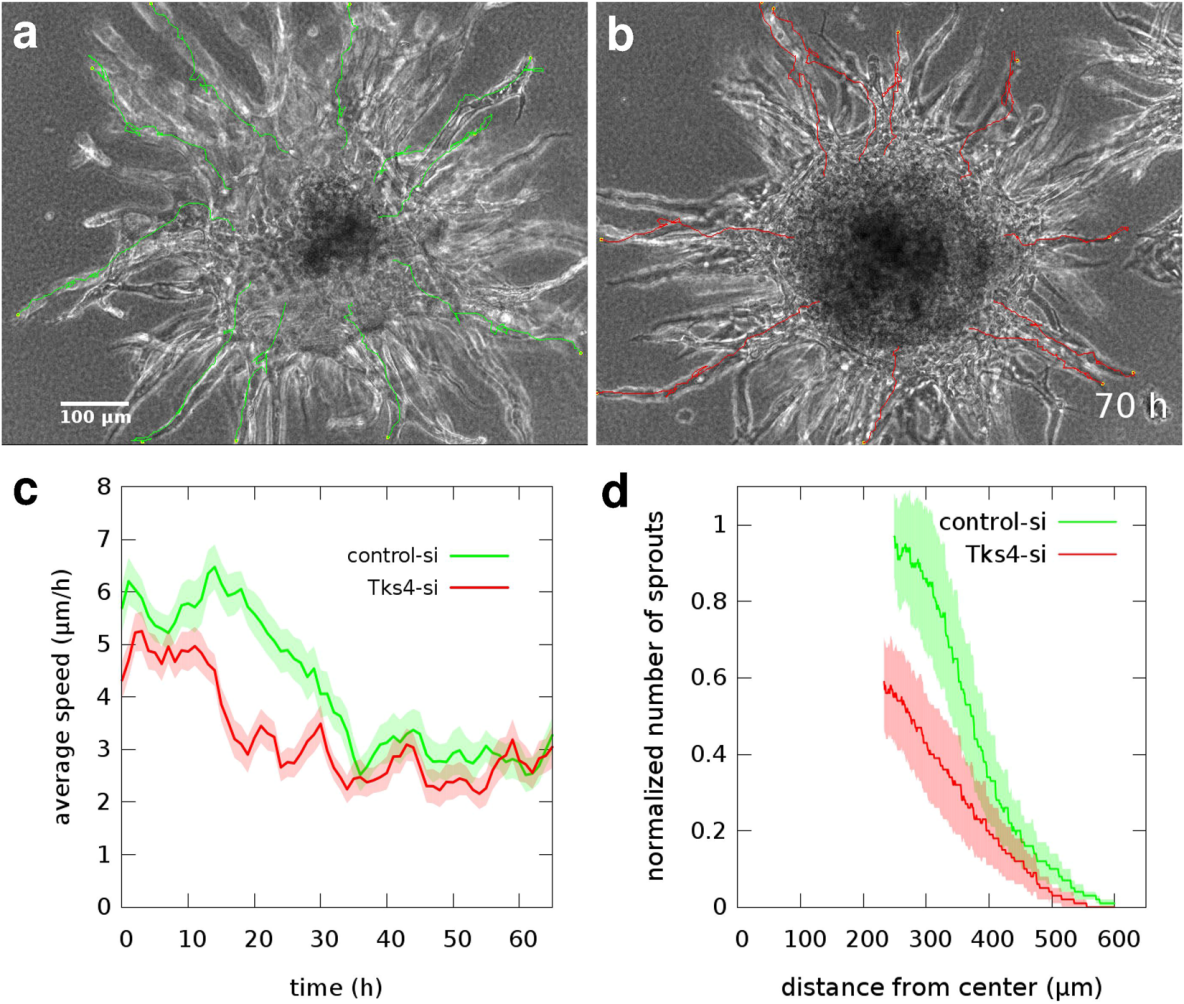
Multicellular sprouting assay utilizing HUVEC aggregates embedded in fibrin gel. Representative phase contrast microscopic images depict sprout arbors of control-siRNA (**a**) or Tks4-siRNA (**b**) transfected cells, after 70 hours in culture. Sprout tip locations are indicated by superimposed trajectories. Scale bar: 100 *μ*m, also see Supplementary Video 7. (**c**) Average sprout tip speeds, as a function of culture time. Data was pooled from 3 independent sets of experiments and includes n = 30 sprout tip trajectories in both groups. (**d**) Distribution of vascular sprouts after 3 days in culture: the number of sprouts in the sprout elongation zone (see Fig. 7) that are longer than the value at the X-axis. Data are pooled from 3 independent sets of experiments with n = 17 control-si (green) and n = 25 Tks4-si (red) aggregates. Sprout numbers were normalized with the average sprout count of control-si aggregates, determined at the inner boundary of the sprout elongation zone. Error stripes correspond to SEM.

Endothelial cell aggregates cultured within ECM gels tend to grow multicellular sprouts and form branching lumenized tubular structures within 2–3 days. This process is considered as an *in vitro* model of angiogenesis^30^. To test the impact of Tks4 on angiogenic sprouting activity, we embedded HUVEC and HMVEC aggregates within fibrin gels and imaged the patterning process by time-lapse videomicroscopy for 3 days (Fig. 6, Supplementary Fig. S6, Supplementary Videos S7 and S8). Again, we compared Tks4-silenced endothelial sprouts with appropriate negative controls transfected with control-siRNA. As a quantitative measure of the patterning process, we calculated the time-dependent average speeds of sprout tips, tracked using the time-lapse image series (Fig. 6c). HUVEC sprout tips initially advance at 5–6 *μ*m/h, and after the first day slow down to speeds 3 *μ*m/h. HMVEC sprouts expand faster: with 8–14 *μ*m/h during the first day, followed by 4–8 *μ*m/h in the second day (Supplementary Fig. S6). However, both Tks4-silenced HUVEC and HMVEC sprouts are slower than sprouts in the negative control cultures.

**Figure 7:**
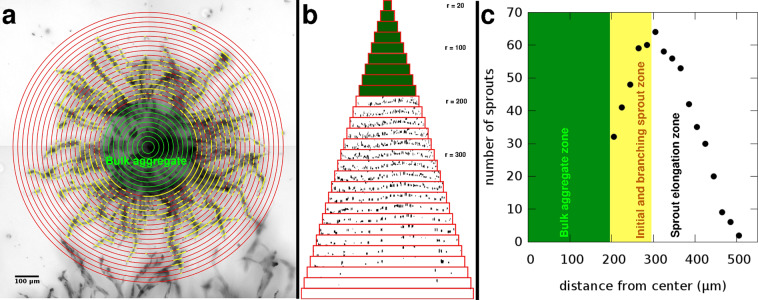
Quantitative analysis of endothelial sprout growth from aggregates. (**a**) A sprout arbor grown in 3 days from a fibrin gel-embedded HUVEC aggregate. The micrograph is a minimum intensity projection of a z-stack of 14 brightfield images covering a 280 *μ*m deep region. Superimposed concentric circles (red) indicate the cylinder surfaces where sprout traversal (yellow) is counted. The initial aggregate is marked by a green circle. Scale bar: 100 *μ*m. (**b**) The concentric cylinders shown in (**a**) – with radius values ranging from 20 *μ*m to 560 *μ*m – are rolled out side-by-side and ordered by their perimeter. Black patches indicate sites where a sprout traversed through the cylinder surface. Green color indicates cylinders within the initial aggregate with no sprouts. (**c**) Number of sprout traversals vs. the distance from the aggregate centre, calculated from data shown in panel (b). At the surface of the aggregate wide sprouts are detected that tend to branch, yielding a characteristic branching zone (yellow). This is followed by the sprout extension zone where sprout numbers decrease as radial distance increases. The boundary between the branching and elongation zones is identified at the radius where the number of sprout traversals is maximal.

After 3 days in culture we quantitatively analyzed the morphology of the sprout arbors developed in the assay. In the vicinity of the aggregate surface, incipient sprouts often branch and create a dense network that we identify as the sprout branching zone. Subsequently, individual sprouts elongate radially within a volume we identify as the sprout elongation zone. We developed an image analysis tool to count the number of sprouts that traverse aggregate-centered cylinders of various radii (see Methods, Fig. 7). In the analysis shown in Fig. 6d, we included only data obtained from the sprout elongation zone. To pool data from independent experiments, we normalized the sprout numbers with the average of control-si sprout counts, determined at the inner boundary of the sprout elongation zone. In accord with the sprout extension speed data, inactivation of Tks4 in both HUVEC and HMVEC aggregates resulted in a 40% reduction in the overall number of sprouts.

### Vessel density in fetal chorion is decreased by knockout of Tks4

**Figure 8:**
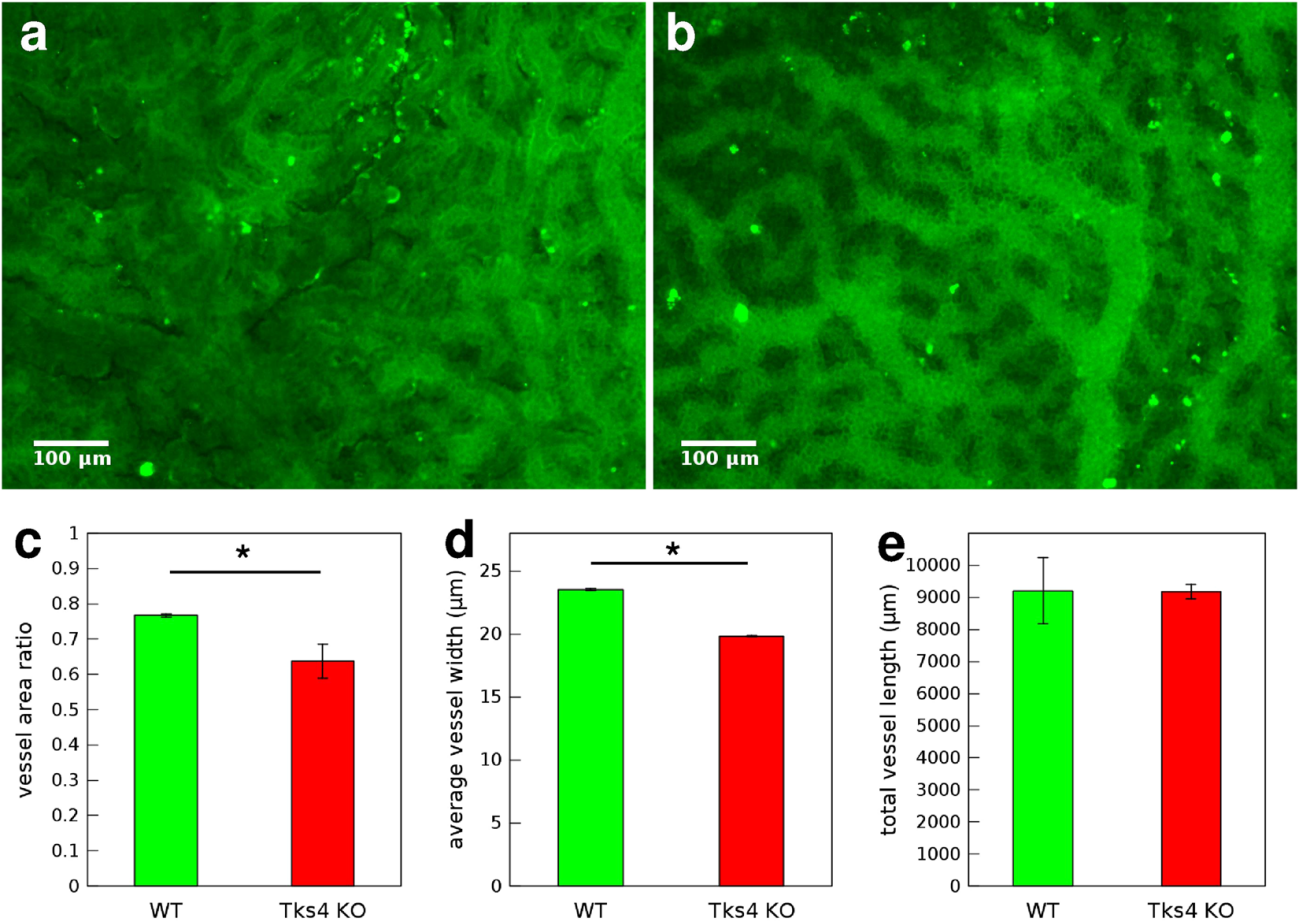
Chorion vessel analysis. Representative fluorescent microscopic images of chorions from (**a**) WT or (**b**) Tks4-KO embryos where endothelial cells were visualized by CD34 immunofluorescent labeling (green), scale bar: 100 *μ*m. Quantitative analysis of vessel parameters in 3 WT and 3 Tks4-KO individuals: (**c**) vessel density, (**d**) average width of vessels, (**e**) total length of all vessels in a 865 *μ*m × 650 *μ*m microscopic field. Error bars correspond to SEM and asterisks (*) indicate statistically significant differences with Student’s t-test, $p < 0.05$.

Inactivation of Tks4 in mice is frequently associated with abnormal growth and defects in the circulatory system^31^. To study how Tks4 is involved in these processes we analyzed the vascular network of the fetal chorion. We compared chorions of mouse embryos aging 10.5 dpc chosen from the Tks4 knockout ‘nee’ strain as homozygote wild type or homozygote Tks4 knockout littermates, verified by genotyping. After isolating the chorions we immunolabeled CD34, specifically identifying fetal endothelial cells and outlining the vessels^5^. We used these specimens to identify and quantitatively analyze vessels. We calculated vessel density as the ratio of vessel area (projection) and total area of the quasi 2-dimensional chorions. Wild type chorions were characterized by high vessel density reaching 75%, whereas Tks4 KO chorions typically had lower vessel density of 65%, the difference being significant with Student’s t-test ($p < 0.05$). Two other parameters of chorion vessels were extracted from the images after image analysis. The widths of vessel segments were significantly lower in Tks4 KO chorions, determined by Student’s t-test ($p < 0.05$), whereas no significant difference was observed between the two groups in the overall length of vessels in the unit areas (865 *μ*m × 650 *μ*m microscopic fields) analyzed (Fig. 8).

## Discussion

### Congenital heart defects frequently present in FTH syndrome

Mutations in the Tks4 gene, causing the human congenital Frank-ter Haar syndrome, frequently manifest in cardiovascular defects accounting for death in early infancy^26^. Other mutations of the Tks4 gene in humans are manifested in the Borrone Dermato-Cardio-Skeletal syndrome where symptoms include mitral valve prolapse^27^. In these patients the two valve flaps of the mitral valve bulge in the left atrium and cannot close smoothly causing floppy valve syndrome (Barlow’s syndrome). Mice lacking the Tks4 gene are characterized by high perinatal lethality, runted growth and cardiac abnormalities^25,31^. Thus, heart defects related to Tks4 mutations point to a role of Tks4 in heart morphogenesis.

Formation of the vasculature in developing vertebrate embryos is of critical importance in early heart morphogenesis. After formation of the primary vasculature during vasculogenesis, the assembly of the midline tubular heart from bilateral precursor fields takes place with the involvement of endothelial cells^8,32–34^. These endothelial cells comprise the endocardial layer, which is continuous with the endothelial lining of blood vessels. Some endocardial precursors undergo epithelial-to-mesenchymal transformation, move into an ECM-rich tissue environment and contribute to formation of the septa and valves of the heart^35^. Thus, the ability of Tks4 to modulate endothelial motility in complex 3D ECM environments, as demonstrated in this paper, point to a possible mechanistic explanation of the congenital cardiovascular defects observed in patients.

### Endothelial invasion relies on secreted MMPs and podosomes

Motility of endothelial cells in 3-dimensional ECM rely on cell surface expression of matrix metalloproteinases (MMPs) specifically cleaving the ECM and contributing to its remodeling. MMPs are recruited to podosomes, protrusion sites with pericellular proteolytic activity, characterized by co-localization of F-actin, cortactin and MT1-MMP^36^. Endothelial invasion through collagen matrices requires MT1-MMP^37,38^, directly cleaving ECM components including collagens, fibronectin and fibrin and proteolytically activating other MMPs^39^. Tks adaptor proteins, Tks4 and Tks5, were identified as important components of podosomes/invadopodia: their absence was demonstrated to result in decreased MMP activity in various cell types including fibroblasts and melanoma cells^19,22^. In line with these results, our data show that if Tks4 is absent, endothelial cells have fewer podosomes and decreased ECM cleavage and similarly to macrophages and fibroblasts they have reduced motility in 3D collagen I matrix. Remarkably, this loss of motility is specific to the 3D culture environment as no negative impact was found in 2-dimensional random motility assays. Thus, decreased 3D motility is likely a consequence of defective matrix cleavage rather than a defect of the intracellular motility apparatus. A similar effect was also reported recently in the C8161.9 human melanoma cell line, which exhibits decreased growth in 3D but not in 2D environments^22^.

### Vascular defects consistent with decreased branch number, not branch length

In sprouting angiogenesis endothelial cells break down the vascular basement membrane and extend multicellular sprouts in the surrounding ECM. Vascular endothelial growth factor (VEGF) was shown to induce the formation of podosomes, directly preceding the formation of new vessels at branching points^40,41^. Absence of Tks4 in endothelial cells in the present study resulted in considerable decrease in the number of sprouts initiated from endothelial aggregates. It also decreased the initial speed of sprout extension, determined by time-lapse video analyses. These findings are indicative of the requirement of both proper localization of MMPs at sites of sprout initiation and sufficient MMP activity during sprout extension, thereby positioning MMPs as permissive agents of multicellular sprouting. In accord with our findings, MT1-MMP was reported earlier to be confined to the sprouting tips of new capillary structures *in vivo*^42^ and playing pivotal role in regulating other MMPs in angiogenesis^28^.

To study the contribution of Tks4 to the development of vascular network *in vivo*, we compared the morphology of fetal chorions of wild type and Tks4 knockout (nee) mice. Chorions from Tks4 knockout embryos exhibited a reduced overall blood vessel density. While Tks4- vessels appeared to be narrower, there was no apparent difference in other characteristic features of vessels such as overall length or number of branching points per unit area. Sparser vasculature in chorions of Tks4 knockout mice may explain higher perinatal lethality observed among homozygote Tks4-KO mice.

### Molecular link between Tks4 and MT1-MMP

Groups that developed elegant *in vitro*, and *in vivo* systems to examine the role of Tks proteins in tumor progression mostly focused on the their role in functional podosomes in various cancer cell lines (recently reviewed in^36^). Downregulation of either Tks4 or Tks5 expression in transformed cells resulted in the loss of functional invadopodia, at least in part due to defects in the re-localization of MT1-MMP into invadopodia-like structures^19^. Importantly, overexpression of Tks5 could not compensate for the Tks4-dependent loss of functional invadopodia. This suggests that Tks4 and Tks5 have non-overlapping functions in regulating proteolytic activity of cancer cells. Depletion of Tks4 in an *ex vivo* chicken chorioallantois membrane (CAM) model or in a murine lung metastasis model was also shown to significantly decrease the number of tumor extravasations and metastases^43^. Reduced number of macrometastases has also been reported in Tks4-defficient human breast cancer and mouse melanoma models^22,43^.

Tumor progression results from a delicate interplay between cancer cells and the tumor microenvironment. In the above described experimental models the “tumor cell-intrinsic” function of Tks4 was investigated. To the best of our knowledge the present work is the first to examine the function of Tks4 in endothelial cells, also regarded as key players of the tumor microenvironment and a limiting factor in tumor progression. Our results strongly suggest a fundamental role of Tks4 in the regulation of endothelial functions and potentially a significant role in tumor-induced angiogenesis.

Extending earlier findings^19^, here we show that similarly to transformed cells, endothelial cells also use a Tks4-dependent mechanism to regulate the localization and activity of metalloproteinases for cleavage of ECM-proteins. Furthermore, impaired movement of Tks4-deficient primary bone marrow-derived macrophages in 3-dimensional collagen gels warrants further characterization of Tks-4 functions in non-transformed cells. Although the exact mechanism by which Tks4 controls the activity of MT1-MMP has not been described, the striking similarity between the phenotypes of Tks4-KO and MT1-MMP-deficient mice strongly suggest that they are part of a common signaling pathway controlling, among others, body size and bone development^44^.

## Methods

### Animals

Tks4-knockout Nee (nose eye ear) mouse strain was obtained directly from The Jackson Laboratory (B10.Cg-H2h4 Sh3pxd2bnee/GrsrJ, stock number: 006446). Mice were kept in ventilated cages under 12-hour dark/12-hour light cycle in the SPF animal facility of Debrecen University and received food and water ad libitum. Specific pathogen free (SPF) status was evaluated every 3 months. Animal procedures in this study were conducted under the approval of the Institutional Animal Ethics Committee (DEMÁB) of Debrecen University (approval number: 8/2014/DEMÁB). The animals were maintained and handled in accordance with Directive 2010/63/EU of the European Parliament and of the Council on the protection of animals used for scientific purposes.

### Cell cultures

Primary endothelial cultures: both human umbilical vein endothelial cells (HUVEC, CC-2519) and human cardiac microvascular endothelial cells (HMVEC-C, CC-7030, lot: 18TL075839) were obtained from Lonza and were maintained in EGM-2 medium (Lonza) for up to 3 passages in tissue culture dishes (Greiner).

Primary macrophage cultures: Murine bone marrow-derived macrophages (BMDM) were obtained from the femurs and tibias of 6–10 weeks old Tks4-deficient (nee) and wild-type mice, as described earlier^45^. Briefly, bone ends were cut off with scissors and the marrow was flushed out with DMEM (Thermo Fisher Scientific) from the bone cavity. Washed marrow cells (in DMEM) were resuspended in red blood cell depletion buffer containing 0.727% NH4Cl-0.017% Tris-Cl (pH7.2)-phosphate buffered saline (PBS). After washing, cells were seeded on low adherence culture dishes (Sarstedt) in DMEM medium containing 10% FBS, 100 IU/ml penicillin G, 100 *μ*g/ml streptomycin (all from Thermo Fisher) which was further supplemented with 10% of CMG14-12 cell culture supernatant, at 0.5 × 10^5^ cells/cm^2^ density. After three days in culture the cells were washed off the plates by vigorous pipetting with 0.02% EDTA in PBS, and the recovered cells were replated under the same conditions in 10 cm suspension culture dishes (Corning) at the density of at 3 × 10^5^ cells/dish. BMDM were harvested after an additional 3–4 days in cultures.

Primary fibroblast cultures: Fibroblasts of Tks4-deficient (nee) and wild-type mice were prepared from tail tips. After 30 minutes of digestion of the tail pieces in 1000 U/ml collagenase (Type XI-S, Sigma) at 37 °C, cells were suspended by pipetting and plated on tissue culture dishes (Corning) in DMEM (Sigma) supplemented with 10% FBS (Gibco) and cultured for several days during which mainly fibroblast cells survived.

All culture media were supplemented with Penicillin-Streptomycin-Amphotericin B (Sigma). Cell cultures were kept at 37 °C in humidified incubator with 5% CO_2_ atmosphere.

Cells in monolayer cultures were briefly incubated with 0.5 mg/ml trypsin and 0.2 mg/ml EDTA (Sigma) in PBS (phosphate buffered saline, 0.1 M phosphate, 0.9% NaCl, pH 7.4, GIBCO) then cells were rinsed and seeded in various dishes and media as required by the specific experimental setup.

For 2-dimensional migration experiments, HUVECs were seeded in EGM-2 medium (Lonza) in tissue culture dishes coated with fibronectin or collagen I. Coating was achieved by pre-incubation with 10 *μ*g/ml fibronectin (Sigma) in PBS for 16 hours or covering with thin 1.7 mg/ml collagen I (Corning) gel layer.

For 3-dimensional migration experiments, cells were suspended in collagen I gel (rat tail collagen I, Corning) prepared according to manufacturer’s gel preparation protocol and in accordance with a protocol described earlier^46^. HUVEC cultures in 1.7 mg/ml collagen I gel were kept in EGM-2 medium supplemented with 40 ng/ml bFGF, 40 ng/ml VEGF (Pierce), 80 nM PMA (Fluka), and 50 *μ*g/ml ascorbic acid (Sigma) as described earlier^47^. Primary mouse fibroblast cultures in 1.7 mg/ml collagen I gel were kept in DMEM (Sigma) supplemented with 10% FBS (Gibco). Primary mouse macrophage cultures in 2.2 mg/ml collagen I gel were kept in DMEM (Sigma) supplemented with 10% FBS (Gibco) and 10% MCSF.

### Gene silencing, siRNA transfection

HUVEC cultures were grown in EGM-2 medium (Lonza CC-3162). Low passage (maximum passage 3) HUVECs were seeded at a density of 1.5 × 10^4^ cells/cm^2^ on 6-well plates, in 2 ml medium/well. At 60–70% cell density cells were transfected with 40 pmol of Tks4-specific siRNA (si3) or control oligonucleotide (si3c), termed Tks4-si or control-si, respectively, in the presence of Lipofectamine 2000 (Invitrogen) as described by the manufacturer. The sequences of the oligonucleotides are available in an earlier publication^20^. At least 48 hours after transfection, HUVECs were used in functional assays. Efficiency of Tks4 silencing was verified by western blot analysis of cell lysates prepared from cultures up to 6 days post transfection.

### Western blotting

HUVEC cells were lysed in RIPA buffer (1% Triton X-100, 0.5% deoxy-cholate and 0.1% SDS) containing protease inhibitors Aprotinin, Leupeptin, Pepstatin, Bestatin (Sigma, 20 *μ*g/ml each) and 1 mM PMSF. Lysates (10 *μ*g) were subjected to SDS-PAGE on 8% gels then transferred to polyvinylidine difluoride membranes (Immobilon-P, Millipore). Membranes were blocked with 3% BSA (Sigma), incubated with the indicated primary antibodies then horseradish peroxidase-conjugated secondary antibodies (Amersham), and developed using enhanced chemiluminescence (ECL West Pico, Pierce). Primary antibodies used for western blotting in this study were: anti-beta actin (Sigma, A1978), anti-Tks4 (1:5000 dilution, SH3PXD2B-specific antibody^20^) anti-Tks5 (1:200 dilution, Santa Cruz, sc-30122, generous gift by Dr. Buday, Biological Research Center, Hungarian Academy of Sciences).

### Gelatin degradation assay

Oregon Green 488-conjugated gelatin from pig skin (Sigma) was dissolved at the concentration of 1 mg/ml in 2% sucrose. Coverslips were coated (in 24-well plates) with 0.2 mg/ml Oregon Green 488-conjugated gelatin at 4 °C for 20 minutes in the presence of 40 *μ*l of 0.5% glutaraldehyde diluted in 1 ml PBS. Coverslips were then washed three times in PBS, at room temperature and incubated with 5 mg/ml NaBH_4_ (dissolved in PBS) at room temperature for 3 minutes. Coverslips were washed as above, sterilized in 70% ethanol for 1 min then dried. After incubating in EGM-2 medium (Lonza) at 37 °C for 1 hour, 2 × 10^4^ HUVECs transfected with Tks4-specific or control oligonucleotides were seeded onto coverslips, and cultured for 20 hours at 37 °C with 5% CO_2_ atmosphere in 24-well plates (Greiner). For a subset of experiments for immunocytochemistry HUVECs were cultured for only 5 hours. Fluorescence of Oregon Green 488-conjugated gelatin is lost upon cleavage. Coverslips were fixed with 1% paraformaldehyde for 10 minutes, washed and mounted on microscopic slides with mowiol mountant and were imaged by fluorescent microscopy.

### Immunocytochemistry

HUVECs were seeded on glass coverslips coated with fibronectin (20 *μ*g/ml, Sigma) or Oregon Green 488-conjugated gelatin (Sigma). After culturing for 4 hours cells were treated with 80 nM PMA (Merck) for 1 hour to induce podosome formation then fixed with 4% paraformaldehyde. After permeabilization with 0.2% Triton X-100 (Sigma, T-9284) non-specific binding sites were blocked with 3% BSA (Sigma, A9418). The following primary antibodies were used: anti-MMP14 (1:20 dilution, R&D, MAB918), anti-cortactin (1:50 dilution, Merck, #05-180). For secondary antibodies we used Alexa Fluor 488 donkey anti-mouse IgG (1:400 dilution, Life Technologies, A21202) or Alexa Fluor 647 donkey anti-mouse IgG (1:400 dilution, Life Technologies, A31571). For fluorescent labeling of F-actin, we used TRITC-conjugated phalloidin (Sigma). Finally, cultures were covered with Fluorescent Mounting medium (Dako, S3023). Cells were imaged by confocal laser scanning microscopy on a Nikon TE300 microscope equipped with a 100x objective (NA = 1.30) using the Bio-Rad MRC-1024 system. TRITC and Alexa Fluor 488 fluorochromes were imaged as red and green, respectively, while Alexa Fluor 647 (far red) emission was imaged as blue color for better visibility in co-labeling with red emitting TRITC fluorochrome. Images were processed using NIH ImageJ software.

### Time-lapse videomicroscopy

Time-lapse recordings were performed on either a Zeiss or a Leica microscope system.

Time-lapse recordings of cell motility experiments were performed on a computer-controlled Leica DM IRB inverted microscope equipped with a Marzhauser SCAN-IM powered stage and a 10x N-PLAN objective with 0.25 numerical aperture and 5.8 mm working distance. The microscope was coupled to an Olympus DP70 colour CCD camera. Cell cultures were kept at 37 °C humidified 5% CO_2_ atmosphere in tissue culture Petri dishes (Greiner) in a stage-top incubator (CellMovie) mounted on the powered stage of the microscope. Stage positioning, focusing, illumination and image collection were controlled by a custom-made experiment manager software on a PC. Phase contrast images were collected consecutively every 10 minutes from several microscopic fields for durations up to 48 hours.

Time-lapse recordings of endothelial sprout growth assays were performed on a Zeiss Axio Observer Z1 inverted microscope with 10x Plan Neofluar objective. The microscope was equipped with a Zeiss AxioCam MRm CCD camera and a Marzhauser SCAN-IM powered stage. Cultures within tissue culture Petri dishes (Greiner) were kept in a stage-mounted incubator (Cell Movie) providing 37 °C and a humidified 5% CO_2_ atmosphere. Stage positioning, focusing and image collection were controlled by Zeiss Axiovision 4.8 software and a custom experiment manager software module. Phase contrast images were collected every hour from several microscopic fields for durations up to 4 days.

Microscopic images were processed using NIH ImageJ software.

### Quantitative analysis of cell motility

Image sequences were analyzed using a custom cell-tracking program (G-track). With this utility, an operator can mark individual cells and record their position into data files. In the following, we denote the position of cell *i* at time *t* as *x*_*i*_(*t*). The motion of cells is often evaluated in terms of average cell displacement, *d*, over a time period $\tau$ as:

1 $${d}^{2}(\tau )={\langle {x}_{i}(t+\tau )-{x}_{i}(t)\rangle }_{i,t}$$

where the average ${\langle \ldots \rangle }_{i,t}$ is calculated over all possible cells *i* and time points *t*.

Time-dependent average cell speed was calculated similarly from cell position data, with a one hour long time lag ${\tau }_{0}=1h$ as

2 $${v}_{i}(t)={\langle \frac{|{x}_{i}(t+{\tau }_{0})-{x}_{i}(t)|}{{\tau }_{0}}\rangle }_{i}$$

where the average ${\langle \ldots \rangle }_{i}$ is calculated over all possible cells.

### Endothelial sprout growth assay

For endothelial sprout growth assay, HUVEC spheroid aggregates were created by seeding cells in aggregation chambers that do not support cell adherence. Aggregation chambers were made by casting liquid 2% agarose (Invitrogen) and allowing it to gelate in a PDMS micromold (3D Petri Dish, Microtissues) containing pillars that define wells with a diameter and depth of 800 *μ*m. After removing from the micromold the agarose gel chambers were equilibrated with culture medium for 2 hours before transferring 2000 cells in suspension into each well. The chambers were then incubated in EGM-2 medium (Lonza) for 1 day allowing cells to self-organise into spheroid aggregates. These aggregates were collected and embedded in 3 mg/ml fibrin gel prepared as described earlier^48^. Briefly, 3 mg/ml human fibrinogen was combined with 200 U/ml aprotinin, 2 U/ml human thrombin, 2.5 mM CaCl_2_ (all from Sigma) and 2 U/ml human factor XIII (CSL Behring), then HUVEC aggregates were added and the solution was transferred and allowed to gelate in circular wells. These 6 mm diameter circular open wells of 50 *μ*l volume were created by filament-deposition (“3D”) printing (Ultimaker V2) of polylactic acid (PLA) well walls in 35-mm tissue culture dishes (Greiner) using a customized technique described recently^49^. Fibrin gels containing the aggregates were covered with 3 ml EGM-2 medium supplemented with 40 ng/ml bFGF, 40 ng/ml VEGF (Pierce), 80 nM PMA (Fluka), and 50 *μ*g/ml ascorbic acid (Sigma) as described earlier^47^ and they were kept at 37 °C in a humidified incubator with 5% CO_2_ atmosphere for time-lapse videomicroscopic observations.

### Endothelial sprout growth analysis

For quantitative analysis of endothelial sprout growth *in vitro* we elaborated an image analysis method as a suitable modification of the Sholl analysis^50^. Endothel spheroid aggregates with multiple sprouts were fixed with 4% paraformaldehyde in PBS and stained with toluidine-blue (1.25 mg/ml in PBS). Images were taken with a Zeiss Axio Observer Z1 inverted microscope with 10x Plan Neofluar objective and Zeiss AxioCam MRm CCD camera. Brightfield microscopic images of the sprout arbors were taken as a z-stack image series with dz = 20 *μ*m slice distance in this optical system having approximately 10 *μ*m depth of field. These stack images were segmented on the basis of local intensity variance, (differentiating low-variance out-of-focus and high-variance in-focus objects), a method yielding more reliable results with our particular images, compared to classic edge detection algorithms (e.g. Sobel or Canny). Based on the binarized 2D images and the optical system’s depth-of-field parameter, we generated voxels and created a 3D reconstruction encompassing the full volume of the sprout arbor. Next, we inserted concentric cylinders with radial distance = 20 *μ*m in the 3D reconstructed volume and recorded the areas where sprout segments traversed the cylinder surfaces. After rolling out and flattening the cylinders in 2D, we applied ImageJ particle detection algorithm^51^ to identify individual sprouts. We used these particle count data to calculate and plot sprout length distribution (Fig. 7). The source code of the algorithm is shared at https://github.com/gulyasmarton/SproutAnalysis/.

### Immunohistochemistry

Chorions from mouse embryos aging 10.5 dpc were isolated and fixed with 4% paraformaldehyde. Immunohistochemistry was performed with a free-floating sample incubation procedure. After permealibization with 1.25% Triton-X100 (Sigma) in PBS, CD34 was detected using anti-CD34 antibody (1:50 dilution, rat monoclonal, MA122646, Life Technologies) and Alexa 488-conjugated anti-rat secondary antibody (2.5 *μ*g/ml, A21208, Life Technologies). Immunolabeled chorions were then mounted on microscopic slides with fluorescent mountant medium (S3023, DAKO). Immunofluorescence was imaged using a Zeiss Axio Observer Z1 microscope equipped with 40x EC Plan-Neofluar objective, Colibri illumination system and AxioCam MRm camera. Images were processed using NIH ImageJ software.

## Supplementary information

Supplementary information

Supplementary Video S1

Supplementary Video S2

Supplementary Video S3

Supplementary Video S4

Supplementary Video S5

Supplementary Video S6

Supplementary Video S7

Supplementary Video S8

## Data Availability

Data generated or analysed during this study are included in this published article and its Supplementary Information files, software tool source codes are available on Github and raw data are available on Open Science Framework.
