# Author Correction: Enhanced endothelial motility and multicellular sprouting is mediated by the scaffold protein TKS4

**DOI:** 10.1038/s41598-020-68536-8

**Published:** 2020-07-20

**Authors:** Elod Mehes, Monika Barath, Marton Gulyas, Edina Bugyik, Miklos Geiszt, Arpad Szoor, Arpad Lanyi, Andras Czirok

**Affiliations:** 1 Department of Biological Physics, Eotvos University, Budapest, Hungary; 2 Department of Immunology, Faculty of Medicine, University of Debrecen, Debrecen, Hungary; 3 First Department of Pathology and Experimental Cancer Research, Semmelweis University, Budapest, Hungary; 4 Department of Physiology, Faculty of Medicine, Semmelweis University, Budapest, Hungary; 5 Department of Biophysics and Cell Biology, Faculty of Medicine, University of Debrecen, Debrecen, Hungary; 6 Department of Anatomy & Cell Biology, Medical Center, University of Kansas, Kansas City, KS USA

Correction to: *Scientific Reports* 10.1038/s41598-019-50915-5, published online 07 October 2019

The original version of this Article omitted an affiliation for Monika Barath. The correct affiliations for Monika Barath are listed below:

Department of Immunology, Faculty of Medicine, University of Debrecen, Debrecen, Hungary

Department of Physiology, Faculty of Medicine, Semmelweis University, Budapest, Hungary

This error has now been corrected in the PDF and HTML versions of the Article.

