# Supplementary material for: Enhanced endothelial motility and multicellular sprouting is mediated by the scaffold protein TKS4
Source: Sci Rep. 2019 Oct 7;9:14363. doi: 10.1038/s41598-019-50915-5 (PMC6779758; doi:10.1038/s41598-019-50915-5)
Supplement: Supplementary file 1 — Supplementary information [file 41598_2019_50915_MOESM1_ESM.pdf]

Supplementary Information

Title: Enhanced endothelial motility and multicellular sprouting is mediated by the scaffold protein TKS4

Authors: Elod Mehes, Monika Barath, Marton Gulyas, Edina Bugyik, Miklos Geiszt, Arpad Szoor, Arpad Lanyi*, Andras Czirok*
* equal contribution

Supplementary Figures

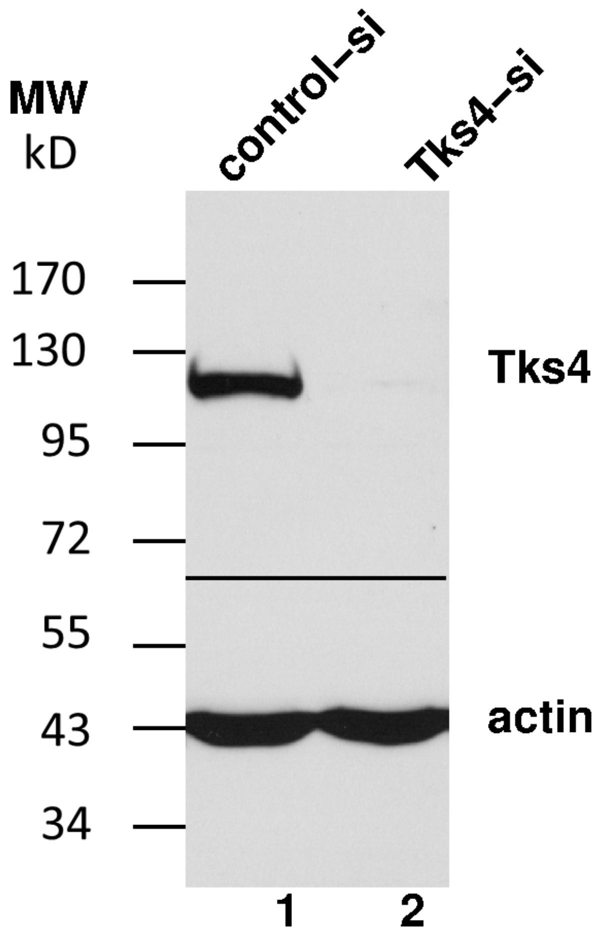

Supplementary Figure S1
Western blot analysis of Tks4 silencing.
Cell lysates prepared from HUVEC cultures 6 days after transfection with either control-siRNA (lane 1) or Tks4-siRNA (lane 2) were immunoblotted. Tks4 protein of ~120 kDa in the upper part of the blot was detected with anti-Tks4 antibody, while in the lower part beta-actin was immunodetected for loading control. Molecular weight markers are indicated on the left. Full-length blot is presented in Supplementary Figure S2.

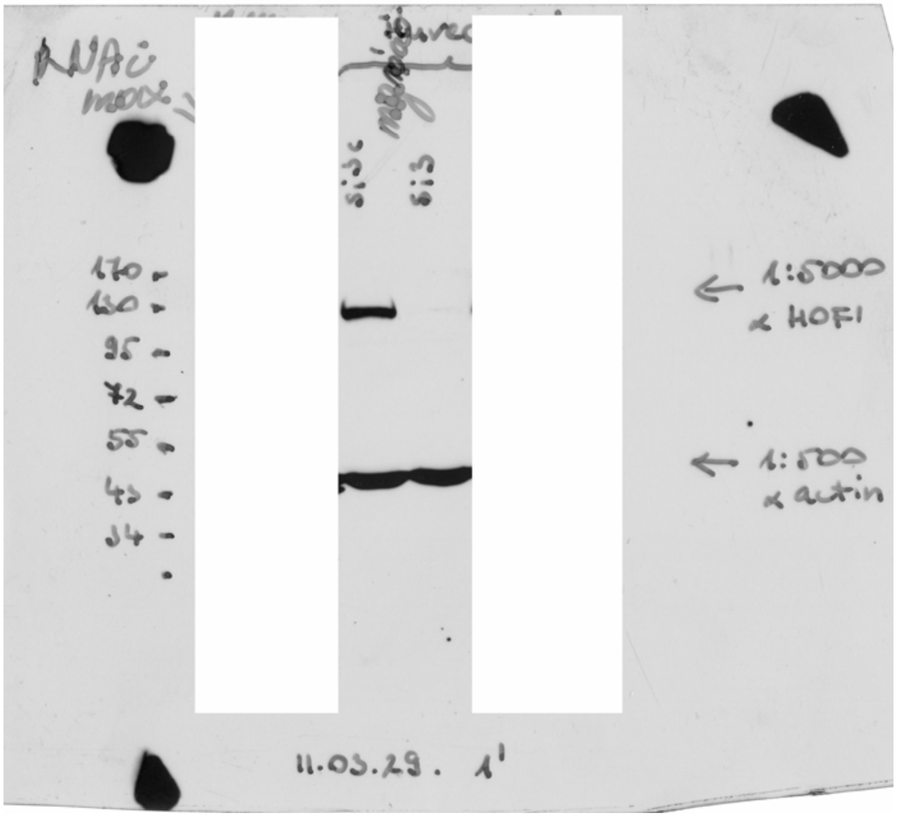

Supplementary Figure S2
Original western blot of Supplementary Figure S1.
Full-length western blot of the immunodetection of Tks4 and actin in cell lysates prepared from HUVEC cultures 6 days after transfection with either control-siRNA (”si3c”) or Tks4-siRNA (“si3”). Tks4 protein of ~120 kDa in the upper part of the blot was detected with anti-Tks4 antibody (“1:5000 a-HOFI”), while in the lower part beta-actin (“1:500 a-actin”) was immunodetected for loading control. Molecular weight markers are indicated on the left.

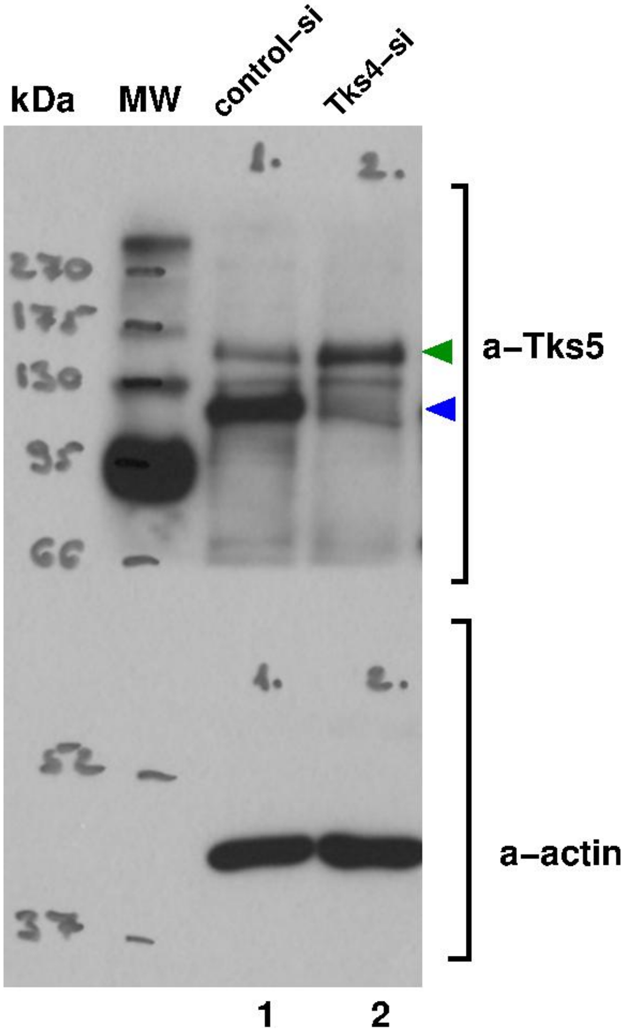

Supplementary Figure S3
Western blot analysis of Tks5 protein after silencing of Tks4.
Cell lysates prepared from HUVEC cultures 2 days after transfection with either control-siRNA (lane 1) or Tks4-siRNA (lane 2) were immunoblotted. Tks5 protein of ~150 kDa in the upper part of the blot was detected by anti-Tks5 antibody (green arrowhead) which also recognized Tks4 protein at ~120 kDa (blue arrowhead). In the lower part of the blot beta-actin was immunodetected for loading control. Molecular weight markers are indicated on the left.

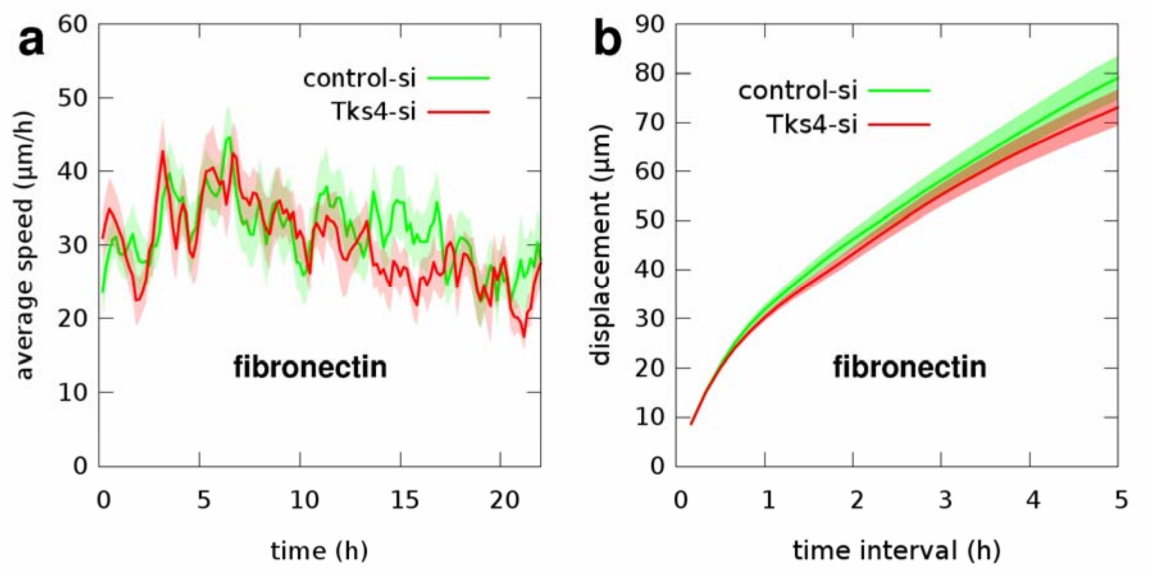

Supplementary Figure S4
Random motility of human cardiac microvascular endothelial cells (HMVEC-C) on 2D fibronectin substrate.
a) Time-dependent cell speeds on fibronectin, averaged over the tracked cells for each time point. b) Average cell displacements on fibronectin during various time intervals. Data in a) and b) were collected from n=20 control-si cells and n=20 Tks4-si cells on fibronectin. Error stripes in graphs correspond to SEM. Also see Supplementary Video S3.

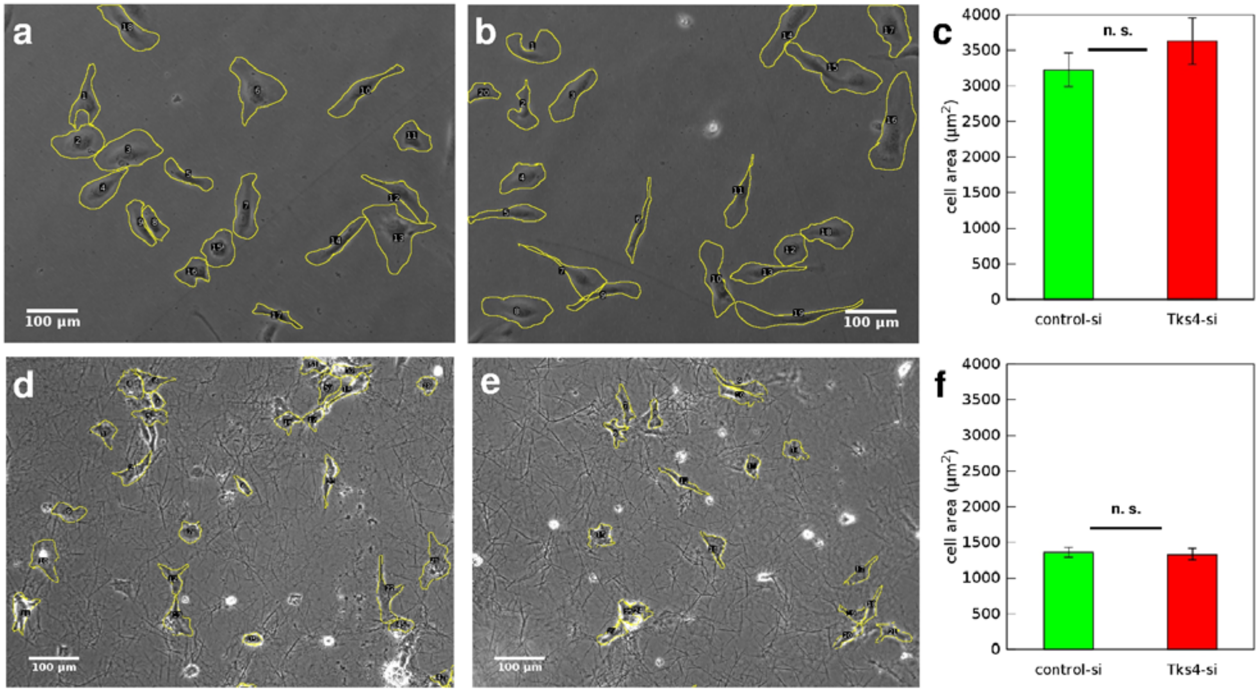

Supplementary Figure S5

Morphology analysis of human umbilical vein endothelial cells (HUVEC) on 2D fibronectin or collagen I substrates.

a-b) Phase contrast images of HUVECs transfected with control-si (a) or Tks4-si (b) RNA on fibronectin-coated plastic. Cells were outlined manually using ImageJ software. c) Quantitative analysis of areas covered by individual cells on fibronectin. Error bars indicate SEM from data of n=39 control-si and n=45 Tks4-si cells, n.s. indicates statistically non-significant differences with Student's t-test, $p<0.05$. d-e) Phase contrast images of HUVECs transfected with control-si (d) or Tks4-si (e) RNA on collagen I-covered plastic substrate. f) Quantitative analysis of areas covered by individual cells on collagen I. Error bars indicate SEM from data of n=49 control-si and n=32 Tks4-si cells, n.s. indicates statistically non-significant differences with Student's t-test, $p<0.05$. Scale bar in a-b, d-e: 100 µm.

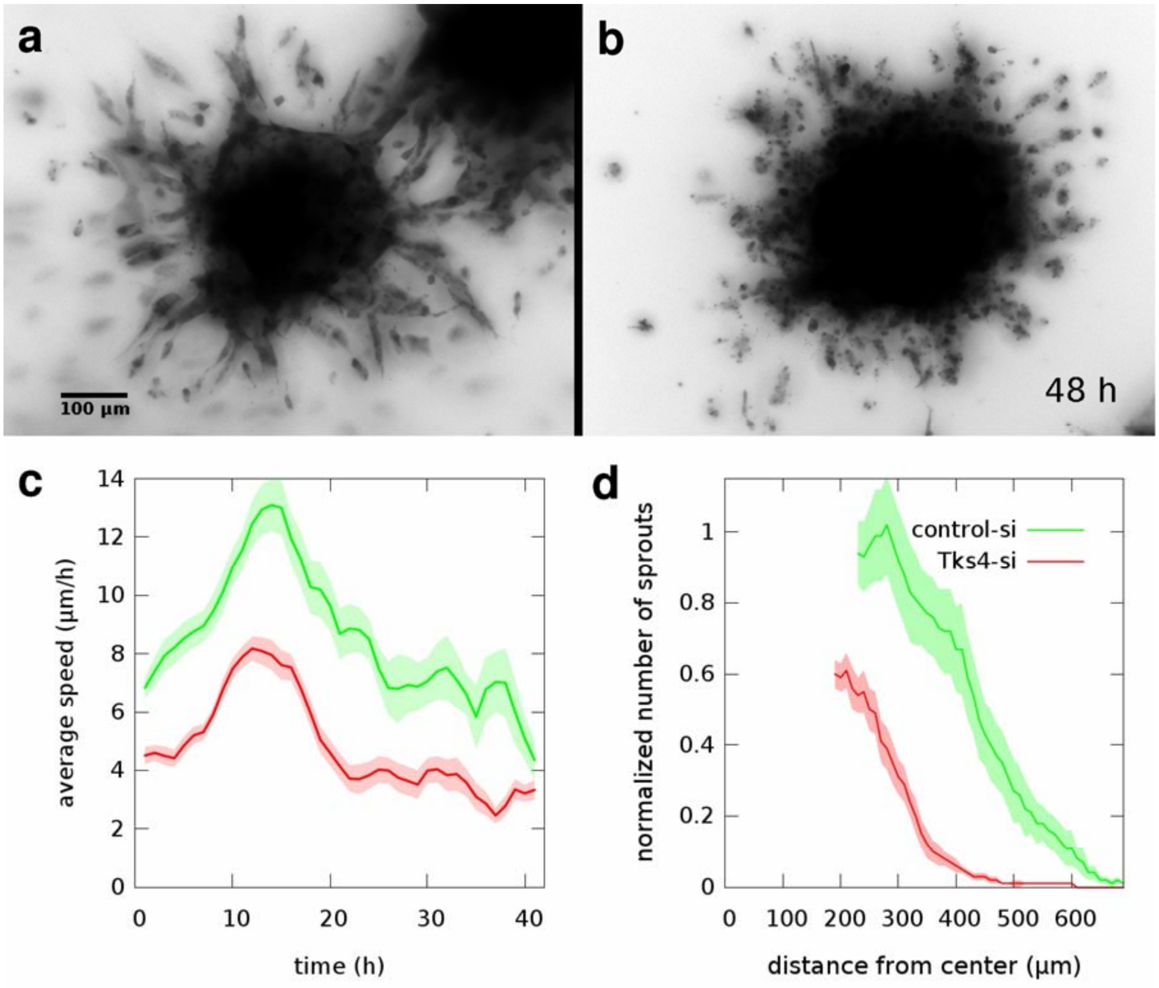

Supplementary Figure S6

Multicellular sprouting assay with human cardiac microvascular endothelial (HMVEC-C) aggregates embedded in fibrin gel.
Representative microscopic images obtained by z-projection of z-stack images show sprout arbors of control-siRNA (a) or Tks4-siRNA (b) transfected cells, after 48 hours in culture. Scale bar: 100 µm, also see Supplementary Video S8. c) Average sprout tip speeds, as a function of culture time. Data was pooled from at least n=23 sprout tip trajectories in both groups. d) Distribution of vascular sprouts after 2 days in culture: the number of sprouts in the sprout elongation zone (see Fig. 7) that are longer than the value at the X-axis. Data are pooled from n=14 control-si (green) and n=12 Tks4-si (red) aggregates. Sprout numbers were normalized with the average sprout count of control-si aggregates, determined at the inner boundary of the sprout elongation zone. Error stripes correspond to SEM.

Supplementary Video captions

Supplementary Video S1

2D random motility of parallel human umbilical vein endothelial (HUVEC) cultures on fibronectin-coated plastic substrate. Cells were transfected with control-siRNA (left panel) or Tks4-specific siRNA (right panel). Phase contrast time-lapse microscopy with 10x objective and 24 h duration.

Supplementary Video S2

2D random motility of parallel human umbilical vein endothelial (HUVEC) cultures on plastic substrate coated with collagen I. Cells were transfected with control-siRNA (left panel) or Tks4-specific siRNA (right panel). Phase contrast time-lapse microscopy with 10x objective and 24 h duration.

Supplementary Video S3

2D random motility of parallel human cardiac microvascular endothelial (HMVEC-C) cultures on fibronectin-coated plastic substrate. Cells were transfected with control-siRNA (left panel) or Tks4-specific siRNA (right panel). Phase contrast time-lapse microscopy with 10x objective and 22 h duration.

Supplementary Video S4

3D random motility of parallel endothelial (HUVEC) cultures in collagen I gel. Cells were transfected with control-siRNA (left panel) or Tks4-specific siRNA (right panel). Note higher motility and the formation of longer cell chains in left panel. Phase contrast time-lapse microscopy with 10x objective and 24 h duration.

Supplementary Video S5

3D random motility of parallel macrophage cultures in collagen I gel. Cells were isolated from wild type (left panel) or Tks4-KO (right panel) mice. Note higher motility in left panel. Phase contrast time-lapse microscopy with 10x objective and 24 h duration.

Supplementary Video S6

3D random motility of parallel fibroblast cultures in collagen I gel. Cells were isolated from wild type (left panel) or Tks4-KO (right panel) mice. Note higher motility in left panel. Phase contrast time-lapse microscopy with 10x objective and 24 h duration.

Supplementary Video S7

Multicellular sprout growth from human umbilical vein endothelial (HUVEC) aggregates in fibrin gel. Cells were transfected with control-siRNA (left panel) or Tks4-specific siRNA (right panel). Note higher sprouting activity in left panel. Phase contrast time-lapse microscopy with 10x objective and 72 h duration.

Supplementary Video S8

Multicellular sprout growth from human cardiac microvascular endothelial (HMVEC-C) aggregates in fibrin gel. Cells were transfected with control-siRNA (left panel) or Tks4-specific siRNA (right panel). Note higher sprouting activity in left panel. Phase contrast time-lapse microscopy with 10x objective and 30 h duration.
